# Identification of Key Immune and Cell Cycle Modules and Prognostic Genes for Glioma Patients through Transcriptome Analysis

**DOI:** 10.3390/ph17101295

**Published:** 2024-09-28

**Authors:** Kaimin Guo, Jinna Yang, Ruonan Jiang, Xiaxia Ren, Peng Liu, Wenjia Wang, Shuiping Zhou, Xiaoguang Wang, Li Ma, Yunhui Hu

**Affiliations:** 1 Tianjin Tasly Digital Intelligence Chinese Medicine Development Co., Ltd., Tianjin 300410, China; tsl-guokaimin@tasly.com (K.G.); tsl-yangjinna@tasly.com (J.Y.); snskrxx0603@163.com (X.R.); tsl-wangwenjia@tasly.com (W.W.); zhousp@tasly.com (S.Z.); 2 State Key Laboratory of Chinese Medicine Modernization, Tianjin 300193, China; liupeng789@tasly.com; 3 Department of Neurology, Tianjin Neurological Institute, Tianjin Medical University General Hospital, Tianjin 300052, China; j3425276900@163.com; 4 Tianjin Medical University Cancer Institute & Hospital, National Clinical Research Center for Cancer, Key Laboratory of Cancer Prevention and Therapy, Tianjin, Tianjin’s Clinical Research Center for Cancer, Tianjin 300060, China; tchwangxg@163.com

**Keywords:** glioma, transcriptome, WGCNA, LASSO, prognostic signature, tumor microenvironment, drug screening

## Abstract

Background: Gliomas, the most prevalent type of primary brain tumor, stand out as one of the most aggressive and lethal types of human cancer. Methods & Results: To uncover potential prognostic markers, we employed the weighted correlation network analysis (WGCNA) on the Chinese Glioma Genome Atlas (CGGA) 693 dataset to reveal four modules significantly associated with glioma clinical traits, primarily involved in immune function, cell cycle regulation, and ribosome biogenesis. Using the least absolute shrinkage and selection operator (LASSO) regression algorithm, we identified 11 key genes and developed a prognostic risk score model, which exhibits precise prognostic prediction in the CGGA 325 dataset. More importantly, we also validated the model in 12 glioma patients with overall survival (OS) ranging from 4 to 132 months using mRNA sequencing and immunohistochemical analysis. The analysis of immune infiltration revealed that patients with high-risk scores exhibit a heightened immune infiltration, particularly immune suppression cells, along with increased expression of immune checkpoints. Furthermore, we explored potentially effective drugs targeting 11 key genes for gliomas using the library of integrated network-based cellular signatures (LINCS) L1000 database, identifying that in vitro, both torin-1 and clofarabine exhibit promising anti-glioma activity and inhibitory effect on the cell cycle, a significant pathway enriched in the identified glioma modules. Conclusions: In conclusion, our study provides valuable insights into molecular mechanisms and identifying potential therapeutic targets for gliomas.

## 1. Introduction

Glioma is a prevalent malignant brain tumor that originates from neuroglial cells within the skull [1]. According to the report summarized in the study of Lin et al. [2], the overall annual incidence of glioma ranges from 2.82 to 7.70 per 100,000 individuals. Specifically, the incidence of non-glioblastoma (non-GBM) varies from 4.80 to 7.70 per 100,000 person-years, while the incidence of glioblastoma multiforme (GBM) ranges from 2.82 to 5.10 per 100,000 person-years. Pathologically, gliomas are typically classified into various types, including astrocytoma, oligodendroglioma, and GBM, with GBM accounting for about 50% of all glioma cases [3]. The World Health Organization (WHO) classifies gliomas into four grades: WHO grade I, II, III, and IV. Grades I and II are considered low-grade gliomas (LGG), while grades III and IV are high-grade gliomas (HGG), with GBM being classified as WHO grade IV [4]. The prognosis and survival outcomes of LGG are significantly better than those of HGG. Histological classification helps us understand the behavior of gliomas, but molecular classification based on molecular features can more accurately distinguish glioma groups. The 2016 World Health Organization Classification of Tumors of the Central Nervous System (2016 CNS WHO) [5], for the first time, uses molecular features in addition to histology to perform the classification of tumors of the central nervous system, including gliomas. The CNS tumor diagnoses should consist of a histopathological name followed by the molecular features, separated by a comma. For example, glioblastoma, IDH-wildtype; astrocytoma, IDH-mutant; Oligodendroglioma, IDH-mutant and 1p/19q-codeleted; diffuse midline glioma, H3 K27M–mutant; and so on [5]. Additionally, key genetic alterations have been reported in gliomas, such as O6-methylguanine-DNA Methyltransferase (MGMT) promoter methylation, mutations in phosphatase and tensin homolog (PTEN) and tumor protein p53 (TP53), amplification of epidermal growth factor receptor (EGFR), and fusion of the MET tyrosine kinase (MET) gene [6,7]. The identified molecular features provide an effective basis for predicting the prognosis of gliomas.

Clinically, the treatment of glioma faces many challenges, including complex tumor locations, high recurrence rates, difficulty in complete resection, and resistance to radiotherapy and chemotherapy. Therefore, finding more effective treatment strategies and developing personalized treatment plans have become important directions in current glioma research. By deepening our understanding of the molecular mechanisms, gene expression characteristics, and the impact of the tumor microenvironment on gliomas, more targeted approaches can be provided for glioma treatment, thereby improving patient prognosis and quality of life.

Several risk factors have been associated with clinical outcomes in glioma patients, including age, tumor grade, Karnofsky performance status (KPS), MGMT status, isocitrate dehydrogenase (IDH) mutation status, 1p/19q codeletion, extent of surgical resection, tumor location or multifocality, and treatment with radiotherapy and chemotherapy [8,9,10]. However, predicting the survival rate of individual patients remains challenging. To more accurately assess the prognosis of glioma patients, researchers have developed hundreds of prognostic models based on prognostic marker genes. However, there are currently no widely accepted and applied prognostic models [11].

In this study, we aimed to construct a free-scale gene co-expression network related to the clinical traits of glioma patients using a dataset from the Chinese Glioma Genome Atlas (CGGA) database. We developed a prognostic model based on 11 genes associated with the cell cycle and immunity, utilizing the CGGA 693 dataset, a transcriptome dataset containing 693 samples. Subsequently, we validated this model using two datasets: the CGGA 325 dataset and another dataset comprising 12 glioma patients with varying overall survival (OS) durations. The CGGA 325 dataset is another transcriptome dataset containing 325 samples from the CGGA database. We explored the close relationships between the risk score model, clinical traits, the tumor microenvironment, immune checkpoints, and the cell cycle. The prognostic model we constructed establishes an independent prognostic model for glioma and provides new insights into the molecular mechanisms of glioma.

## 2. Results

### 2.1. Identification of Four Modules Significantly Associated with Clinical Traits in Glioma

**Figure 1:**
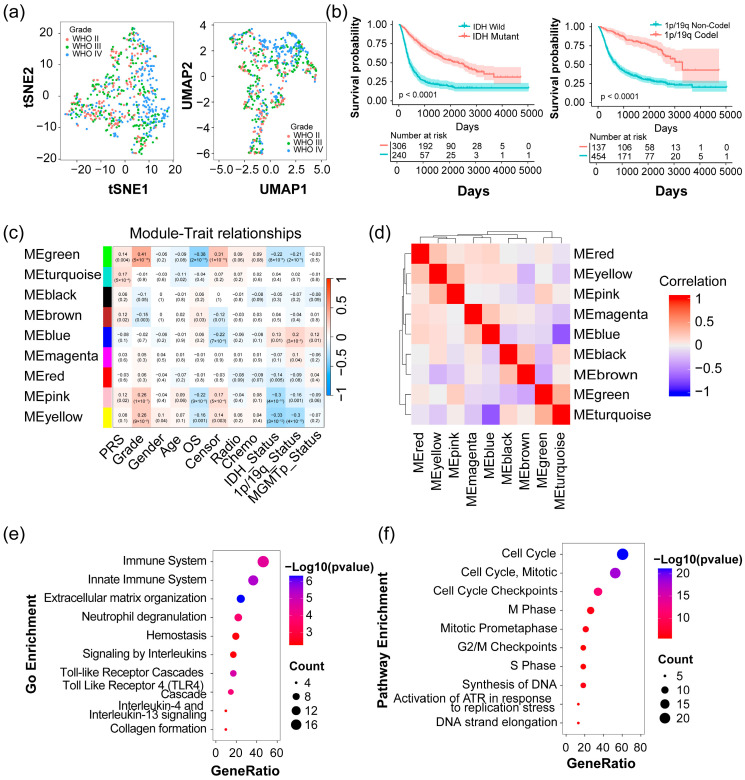
Identification and analysis of glioma-related modules by the weighted correlation network analysis (WGCNA) analysis based on the CGGA 693 dataset. (**a**) t-distributed stochastic neighbor embedding (tSNE) (left) and uniform manifold approximation and projection (UMAP) (right) clustering of 626 glioma samples from the CGGA 693 dataset based on their cancer grades by using the trimmed mean of M-values (TMM) normalized expression profiles; (**b**) Kaplan–Meier survival analysis for 626 of glioma patients based on IDH status (Wild or Mutant) (left), and 1p/19q status (Non-codel or Codel) (right); (**c**) Pearson correlation between gene modules identified by WGCNA and clinical traits in glioma patients. Each cell contains the corresponding correlation and *p*-value; (**d**) analysis of Pearson correlation among modules identified by WGCNA; (**e**) pathway enrichment analysis of reactome for core genes identified by the MCODE, a plugin in Cytoscape software, in the yellow and pink modules, and (**f**) that of the green module.

We acquired the raw expression matrix of 693 glioma samples from the Chinese Glioma Genome Atlas (CGGA) database. After the trimmed mean of M-values (TMM) normalization, sample clustering was performed using t-distributed stochastic neighbor embedding (tSNE) and uniform manifold approximation and projection (UMAP) with default parameters, respectively. Consistent clusters were obtained by the two methods, and the results are depicted in Figure S1a–c. The individual tumor sample was color-coded based on their respective clinical attributes, such as glioma grade, Primary-Recurrent-Secondary (PRS) types, and histology types. This analysis revealed two clusters: a major group comprising 626 samples and a small group consisting of 67 samples. While the samples in the smaller group were more concentrated in WHO grade IV glioma and primary types, there was no significant difference observed in the Kaplan–Meier survival analysis between the two groups (Figure S1d). For more precise data, the samples in the major group were selected for further analysis. Figure 1a illustrates the clustering of samples within the major group using tSNE and UMAP. Both methods demonstrate highly consistent results, revealing that different grades of glioma samples have a gradually separated trend as the grade increases from WHO II to WHO IV (Figure 1a). Additionally, Kaplan-Meier survival analysis indicated that samples with isocitrate dehydrogenase (IDH) mutation or 1p/19q codeletion exhibited significantly more favorable prognostic outcomes compared to samples with wild-type variants in the CGGA 693 dataset (Figure 1b). Samples categorized as WHO grade IV, commonly known as glioblastoma multiforme (GBM), exhibit a poorer prognosis in contrast to samples classified as WHO grades II and III (referred to as non-GBM) in CGGA 693 dataset (Figure S1e). These results are in alignment with the current understanding of glioma [12,13].

A total of 5476 uniquely glioma-associated genes were derived from the DisGeNET (https://www.disgenet.org/ (accessed on 17 November 2023)) and GeneCards (https://www.genecards.org/ (accessed on 17 November 2023)) database (Table S1). These genes were utilized in constructing a co-expression network using the weighted correlation network analysis (WGCNA) algorithm with a soft thresholding power of *β* = 10 across 413 high-quality samples, which were obtained by removing those with missing clinical data from the major group of 626 glioma samples (Figure S2a). This network consisted of 9 modules or communities of proteins interconnected by their co-expression patterns (Figure 1c and Figure S2b). After combining clinical traits, four significantly correlated modules were pinpointed. Among these, modules colored in pink, yellow, and green displayed negative correlations with IDH (Pearson *p*-value = 4 × 10^−10^, 3 × 10^−12^, and 8 × 10^−6^, respectively) or 1p/19q status (Pearson *p*-value = 0.001, 4 × 10^−10^ and 2 × 10^−5^, respectively), while the blue module exhibited a positive correlation (Pearson *p*-value = 0.01 and 3 × 10^−5^, respectively). Coincidentally, these identified modules also exhibited reasonable Pearson correlations with other clinical traits such as glioma grade, overall survival (OS), and censor (alive = 0; dead = 1) (Figure 1c). Analysis of Pearson’s correlation between the modules indicated that the identified modules are relatively independent of each other, except for a notably strong correlation between the pink and yellow modules (Figure 1d). After applying filtering criteria with a threshold of module membership (MM) > 0.8 and gene significance (GS) > 0.2 for IDH status, a total of 96 candidate genes were identified, including 11 genes in the pink module, 31 in the yellow module, 38 in the green module, and 16 in the blue module (Figure S2c). Subsequently, a protein–protein interaction network was constructed with the STRING database (https://string-db.org/ (accessed on 4 March 2024)), and gene clusters were identified by the plugin molecular complex detection (MCODE) in Cytoscape software (v3.1.0) [14]. Notably, the top three clusters identified by MCODE scores corresponded to the green, blue, and yellow modules in WGCNA, respectively. Additionally, genes in the cluster with the fourth highest score aligned with two modules, the yellow and pink modules, potentially due to the strong correlation between these two modules (Figure S2d, Table S2), demonstrating the robustness of the protein modules identified by the WGCNA algorithm. By analysis of pathway enrichment for MCODE clusters, it was observed that genes from pink and yellow modules were enriched in the immune system, innate immune system, extracellular matrix organization, and neutrophil degranulation (Figure 1e), while genes from green modules correlated more strongly with cell cycle, including cell cycle-mitotic, cell cycle checkpoints, etc. (Figure 1f). The MCODE cluster corresponding to the blue module mainly correlated with ribosome biogenesis. For clarity, we defined the pink and yellow modules as the unified Immune module, the green module as the Cell cycle module, and the blue module as the Ribosome module in the subsequent content. These results suggest that the biological functions or processes represented by these co-expression modules may potentially play significant causal roles in the initiation or progression of glioma.

**Figure 2:**
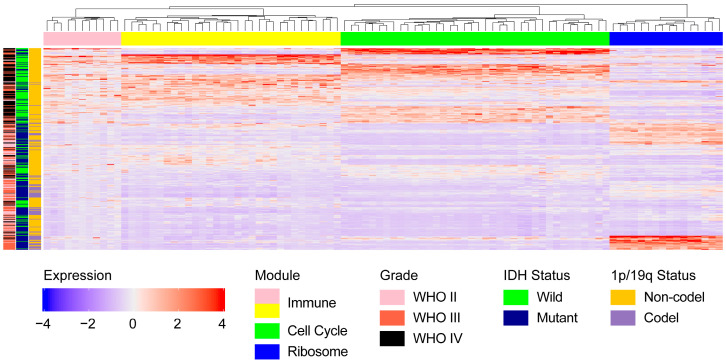
Heatmap depicting the expression of 96 candidate genes in four modules significantly associated with glioma and their relationship with clinical traits including grade (including WHO II, WHO III, and WHO IV), IDH status (Wild or Mutant type), and 1p/19q status (Non-codel or Codel type) in 413 high-quality glioma samples, which were obtained by removing those with missing clinical data from the major group of 626 glioma samples; the four modules were represented by their primary biology function.

Kaplan–Meier survival analysis showed that the majority of genes in the Immune and Cell cycle modules, which are expressed at lower levels in glioma, are associated with a more favorable prognosis compared to genes expressed at higher levels, while the opposite trend was observed in the Ribosome module (Table S3). This finding was further supported by the expression of the module genes in patients, where reduced expression was noted in the Immune and Cell cycle modules in patients with IDH or 1p/19q mutations, typically associated with WHO II or WHO III glioma grades and a more favorable prognosis (Figure 2).

Conversely, a contrasting pattern was observed in the Ribosome module (Figure 2), where most genes displayed higher expression levels in patients with a favorable prognosis (Table S3). Additionally, a significant positive correlation was identified between the gene expression within the module and the 1p/19q status in glioma patients in the CGGA 693 dataset, indicating that individuals with a 1p/19q codeletion are more likely to display elevated gene expression levels (Figure 2 and Figure S3a). Meanwhile, the correlation with 1p/19q status was also confirmed in the CGGA 325 dataset, where 7 out of 16 candidate genes were found to be highly expressed in patients with a 1p/19q codeletion (Figure S3b).

### 2.2. Construction and Evaluation of Risk Score with the CGGA 693 Dataset

**Figure 3:**
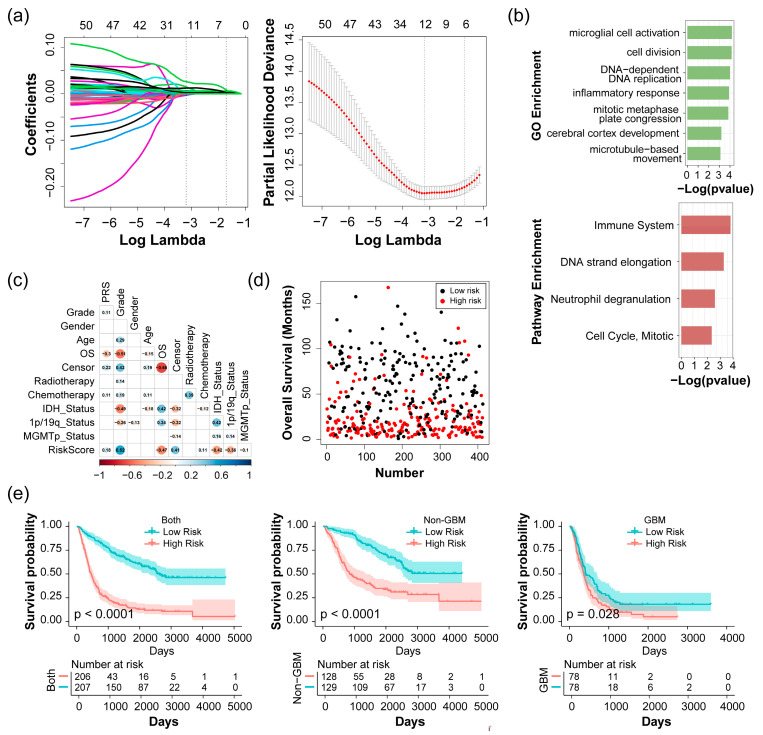
Identification of 11 key prognostic genes by the least absolute shrinkage and selection operator (LASSO) analysis and risk score calculation based on the 413 high-quality glioma samples. (**a**) Coefficient profiles (left, different colors represent different genes.) and 10-fold cross-validation results (right) of LASSO; (**b**) gene ontology (GO) enrichment analysis (top), and reactome pathway enrichment analysis (bottom) for 11 genes determined by LASSO analysis; (**c**) the Spearman correlation coefficient between risk score and clinical traits in glioma patients. Value in each cell represents a correlation between traits, and the red color indicates negative correlations, with blue indicating positive correlations; (**d**) survival time for glioma patients with high risk (red dots) and low risk (black dots); (**e**) Kaplan–Meier survival curves of different groups in 413 glioma samples based on the risk scores-all samples group (left), 257 non-GBM samples group (including WHO II and WHO III for grade, middle) and 156 GBM samples group(including WHO IV for grade, right); The patients with risk score greater than the median of risk scores of all patients were considered as high risk; otherwise, they are considered low-risk patients.

Given the consistent correlations observed between IDH and 1p/19q status and the four significantly identified modules, we applied a filtering process to select module genes with a threshold of MM > 0.8 and GS > 0.2 for both IDH and 1p/19q status (Figure S2c). A total of 69 genes were identified and considered as candidate genes, of which 53 genes belong to the Immune and Cell cycle modules. Subsequently, a least absolute shrinkage and selection operator (LASSO) Cox regression analysis was implemented based on the 53 genes to establish the prognostic signature for glioma patients, identifying 11 key genes (Figure 3a): PLOD1, CCR5, CTSZ, ITGB2, TLR2, ASPM, GINS4, KIF14, KIF2C, KPNA2, and POLD3. Gene ontology (GO) enrichment analysis in the biological process (BP) revealed that many of these genes are linked to glioma-related pathways, particularly enriched in processes such as microglial cell activation, cell division, DNA-dependent DNA replication, inflammatory response, etc. (Figure 3b). The enriched reactome pathways are associated with the immune system, neutrophil degranulation, and cell cycle (Figure 3b). Subsequently, we established a comprehensive risk score consisting of 11 key genes as the glioma-related prognostic signature. The risk score was calculated as follows: expression of PLOD1 * 0.0035 + expression of CCR5 * (−0.0030) + expression of CTSZ * (−0.0003) + expression of ITGB2 * (−0.0010) + expression of TLR2 * 0.0148 + expression of ASPM * 0.0052 + expression of GINS4 * 0.0261 + expression of KIF14 * 0.0041 + expression of KIF2C * 0.0025 + expression of KPNA2 * 0.0067 + expression of POLD3 * 0.0121. For each patient in the CGGA 693 dataset, we computed the corresponding risk score and correlated it with clinical traits based on the Spearman test (Figure 3c). The findings indicate positive correlations between risk score and WHO grade, along with negative correlations with overall survival, IDH status, and 1p/19q status. Specifically, patients with high-risk scores tend to have higher WHO grades and are more likely to exhibit IDH wild type or 1p/19q non-codeletion, which are typically associated with a poor prognosis (Figure 3c,d). By utilizing the median risk score to classify glioma samples into high and low-risk groups, Kaplan–Meier survival curves revealed that glioma patients in the high-risk score group are significantly associated with poor prognosis across all groups in the CGGA 693 dataset, including all glioma patients, the non-GBM group (WHO II and WHO III) and the GBM group (WHO IV) (Figure 3e). Collectively, these results indicated that the calculated risk scores are closely associated with clinical traits and exhibit robust predictive potential for glioma patients.

### 2.3. Analysis of Risk Score as an Independent Prognostic Signature 

**Figure 4:**
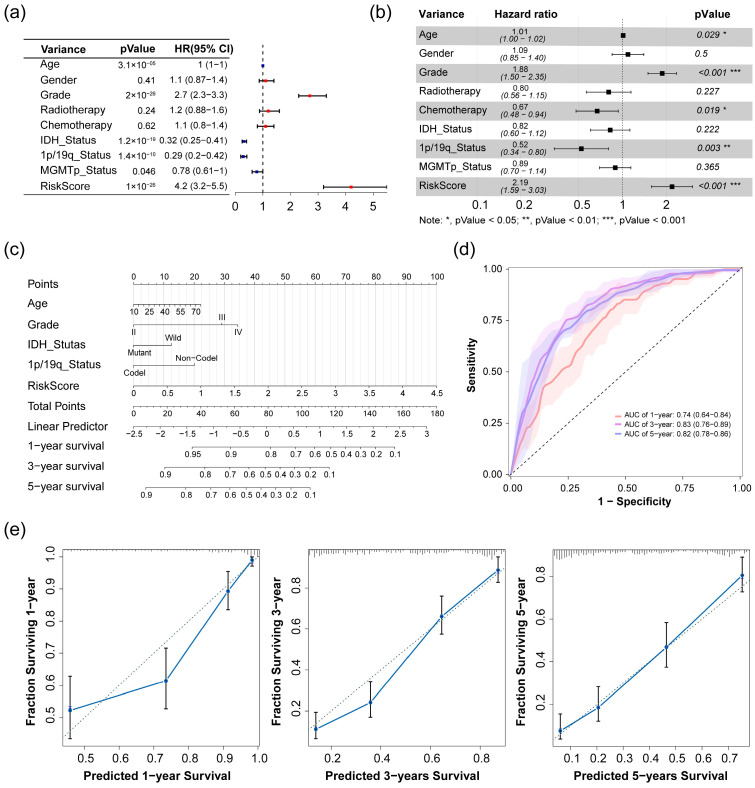
Analysis and validation of prognostic signature in the CGGA 693 dataset. (**a**) Univariate and (**b**) multivariate Cox regression analyses of the association between clinic traits and overall survival (OS) of patients; (**c**) construction of a nomogram model for survival prediction; (**d**) ROC curves with confidence bands (±1 standard deviation, SD) showing the predictive value of the nomogram model for 1-, 3-, and 5-year survival rates; (**e**) calibration plots of the nomogram for 1-year, 3-year and 5-year survival. The X-axis represents the nomogram-predicted probability, and the Y-axis shows the observed probability.

Univariate and multivariate Cox regression analyses of risk scores for glioma were performed in the CGGA 693 dataset. The univariate Cox regression analysis revealed a statistically significant correlation between the risk score and overall survival (OS) (HR = 4.2, *p*-value = 1 × 10^−26^) (Figure 4a). Following adjustment for confounding factors in the multivariate Cox regression analysis, the risk score continued to demonstrate its potential as an independent prognostic indicator for OS in glioma patients (HR = 2.19, *p*-value < 0.001) (Figure 4b). These findings suggest that the risk score serves as an independent prognostic factor for overall survival in patients with glioma.

Subsequently, we developed a nomogram prediction model integrating the risk score and four clinical traits (Age, Grade, IDH status, and 1p/19q status) to forecast clinical outcomes (Figure 4c). To evaluate the predictive efficacy of the nomogram, we performed a receiver operating characteristic (ROC) analysis (Figure 4d) and generated calibration curves (Figure 4e). The calibration curves demonstrated excellent concordance between the predictions and observations at 1-year, 3-year, and 5-year intervals, with the area under the ROC curve reaching 0.74 (±1 standard deviation, SD: 0.64–0.84) at 1-year, 0.83 (±1 SD: 0.76–0.89) at 3-year, and 0.82 (±1 SD: 0.78–0.86) at 5-year, respectively. These results signify a highly predictive performance in the model.

### 2.4. Validation of Prognostic Signature in Glioma Patients with Varying Severity

**Figure 5:**
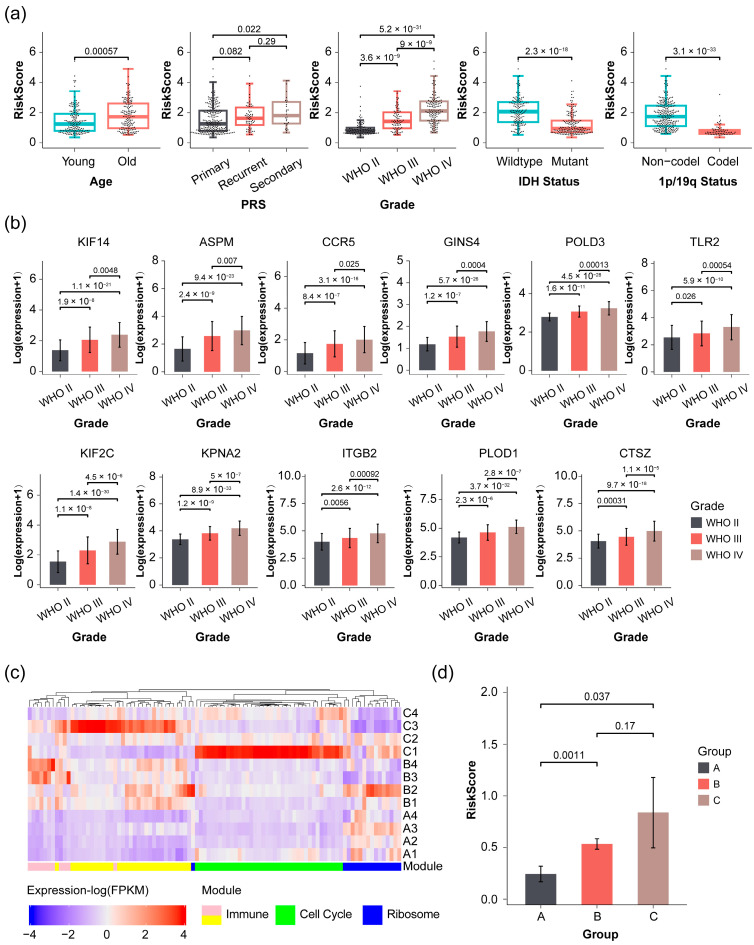
Validation of risk score and 11 prognostic genes in CGGA 325 dataset and a cohort of 12 glioma patients. (**a**) The distributions of the risk score in the different groups of CGGA 325 patients in terms of each clinical trait, such as age, Primary-Recurrent-Secondary (RPS) type, grade, IDH status and 1p/19q status; (**b**) the distributions of expression of 11 identified prognostic genes among different levels of grades patients in the CGGA 325 dataset; (**c**) heatmap of expression for 96 candidate genes in three identified biology modules (Immune, Cell cycle and Ribosome modules, respectively); (**d**) the distribution of risk score in different groups for 12 glioma patients. A group represents low-grade glioma (LGG) patients, and both B and C groups represent glioblastoma (GBM) patients. Among the three groups, the patients in the A group have the longest overall survival (OS), followed by the B group, and group C has the shortest OS. Each group includes four patients. Statistical significances among different groups were determined using the Wilcox test, and a *p*-value < 0.05 was considered significant.

Our prognostic signature was validated in two additional datasets: the CGGA 325 dataset and a cohort comprising 12 glioma patients with diverse prognoses and tumor types. Initially, we assessed the correlations between the risk scores of individual patients in the CGGA 325 dataset and their respective clinical traits, including age (categorized as young or old based on median age), tumor type, grade, IDH mutant status, and 1p/19q codeletion status (Figure 5a). The results revealed significant positive correlations between the risk scores of glioma patients and their age and grade, as well as significant negative correlations with IDH and 1p/19q status. While no statistical differences were observed between primary and recurrent tumors or between recurrent and secondary tumors, it was observed that the risk scores of patients increased with the severity of tumor recurrence. These findings suggest strong correlations between the risk score and the clinical traits of patients within the CGGA 325 dataset. Kaplan–Meier survival also revealed that glioma patients in the high-risk score group are significantly associated with poor prognosis across all sample groups and non-GBM group (WHO II and WHO III) in this dataset (Figure S4a,b), which is consistent with that of in CGGA 693 dataset. However, there was no significant difference in prognosis between high-risk and low-risk groups for the GBM group (Figure S4c), potentially due to the smaller sample size as the number of follow-up days increased (with only four samples remaining after 3000 days of follow-up). Furthermore, based on the LGG and GBM datasets from the Cancer Genome Atlas (TCGA), consistent results were obtained (Figure S4d,e). It was noteworthy that the expression levels of the 11 prognostic genes exhibited statistically significant positive correlations with glioma grade, with levels increasing as the tumor grade advanced (Figure 5b). This finding further corroborates previous results, indicating that patients with lower expression levels of these genes trend to have a more favorable prognosis. For the purpose of organization, we categorized the 12 glioma patients into three distinct groups labeled A, B, and C. Group A comprised four patients diagnosed with LGG who demonstrated a longer OS ranging from 72 to 132 months across all groups. Groups B and C each consisted of 4 patients with GBM. In comparison to group B, where the OS ranged from 40 to 76 months, patients in group C displayed a poorer prognosis, with an OS ranging from 4 to 8 months.

The candidate genes identified within the Immune and Cell cycle modules exhibited increased expression levels in groups B and C compared to group A. In contrast, the blue module exhibited a reverse pattern, showing decreasing expression levels in groups B and C relative to group A (Figure 5c), which is consistent with findings from the CGGA 693 dataset. Furthermore, as the OS of patients decreased, there was a gradual rise in risk scores, indicating a statistically significant negative correlation (Spearman R = −0.71, *p*-value = 0.01) between risk scores and OS across all patients (Figure 5d). We also selected 5 out of the 11 key genes for immunohistochemical validation in these cohorts (Figure S5). Overall, these findings confirm the reliability of the prognostic signature in glioma patients.

### 2.5. Correlation of Risk Score with Immunological Function Analysis in Glioma Patients

**Figure 6:**
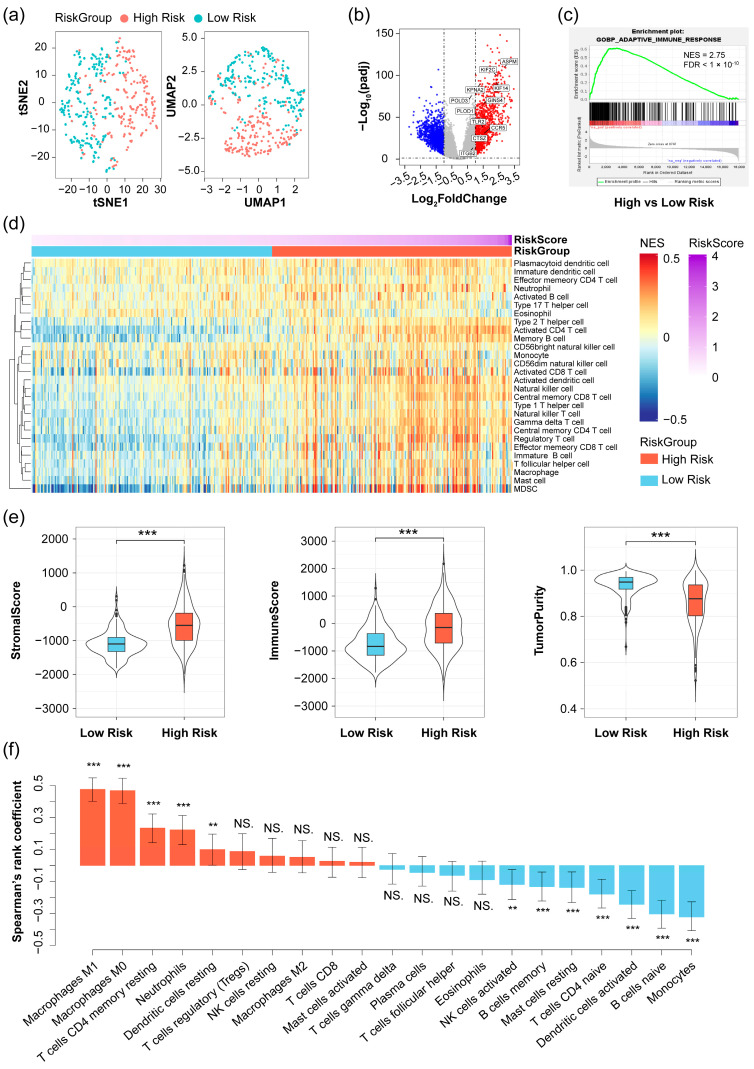
Analysis of the relationship between risk score and tumor microenvironment in the 413 glioma samples from the CGGA 693 dataset. (**a**) tSNE (left) and UMAP (right) plot of 413 samples based on their gene expression, colored by risk group of samples (high risk is defined as a patient with a risk score greater than the median risk score, and low risk is the opposite); (**b**) volcano plot of high-risk versus low-risk group (The red dots represent up-regulated genes, and the blue ones down-regulated genes), in which 11 prognostic genes were marked; (**c**) GSEA plot of high-risk patients compared to low-risk patients in term of adaptive immune response (NES = 2.75, FDR < 1 × 10^−10^); (**d**) heatmap showing the relative abundance of 28 infiltrating immune cell types; (**e**) Violin plots showing the distribution of stroma score, immune score or tumor purity in the low risk and high-risk patients (Wilcox test; ***, *p*-value < 0.01; (**f**) the Spearman’s rank correlation with 95% confidence intervals (CIs) between immune infiltrating cells by CIBERSORTx and risk scores (***, *p*-value < 0.01; **, *p*-value < 0.05; NS., no significance), calculated using the JASP tool.

To investigate the distinctions between patients classified as high risk and low risk, we conducted a cluster analysis on the gene expression of 413 glioma samples from the CGGA 693 dataset previously selected for WGCNA analysis. This analysis was carried out by using tSNE with default parameters, except for the perplexity set to 10, and the UMAP method with default parameters. The results revealed a clear differentiation between patients with high-risk scores and those with low-risk scores (Figure 6a). Differential expression analysis comparing high-risk and low-risk score patients showed significant upregulation of the 11 identified prognostic genes in patients with high-risk scores (Figure 6b). Interestingly, genes upregulated in patients with high-risk scores were significantly enriched for adaptive immune response in the gene set enrichment analysis (GSEA) analysis (NES = 2.75, FDR < 1 × 10^−10^) (Figure 6c), which is consistent with pathway enrichments observed for the 11 prognostic genes (Figure 3b). These findings suggest a correlation between the risk score for glioma patients and immune function.

To investigate the relationship between risk score and tumor immunity, we assessed immune heterogeneity between patients with high and low-risk scores using a single-sample gene set enrichment analysis (ssGSEA). The resulting heatmap displayed the relative abundance of 28 infiltrating immune cells in each glioma patient (Figure 6d). Overall, patients with high-risk scores exhibited a higher degree of immune cell infiltration, indicating an immune-hot phenotype, while those with low-risk scores displayed lower levels of immune cell infiltration, suggesting an immune-cold phenotype, consistent with previous study findings [13,15]. In addition, we also calculated the stromal and immune scores for each glioma patient. The results indicated positive correlations between both stromal and immune scores with the risk score, while tumor purity showed a negative correlation (Figure 6e). These results suggest that the infiltrating immune cells in gliomas increase with rising risk scores, and patients with high-risk scores exhibit an immune-hot phenotype.

It is widely recognized that a high level of immune infiltration tends to be associated with a favorable prognosis [16,17]. However, in this study, there appears to be a contradiction between a high-risk score and an immune-hot phenotype. To address this, we further assessed the proportions of 22 immune cells in each glioma patient using the online software CIBERSORTx (https://cibersortx.stanford.edu/ (accessed on 2 April 2024)), which estimates the abundances of different cell types within a mixed cell population based on gene expression data.

**Figure 7:**
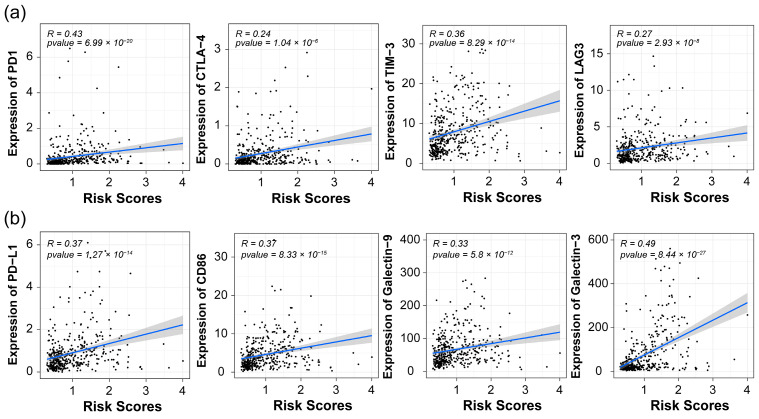
The Spearman correlation between risk score and immune checkpoint proteins. The upper panel (**a**) shows immune checkpoint receptors, including PD-1, CTLA-4, TIM-3, and LAG3, and the lower panel (**b**) shows corresponding ligands for each receptor.

Upon conducting a Spearman correlation analysis between risk scores and the proportions of the 22 infiltrating immune cells, we observed that the infiltrating cells in patients with high-risk scores were predominantly associated with immune suppression, such as macrophage, regulatory T cell, and neutrophil, etc. (Figure 6f). Moreover, the risk score exhibited significant positive correlations with well-known checkpoints and their corresponding ligands (Figure 7a,b, Spearman test). It is well-documented that overexpression of immune checkpoints in tumors is linked to a poor prognosis [18,19,20]. In summary, the high proportion of immune suppression cells and high expression of checkpoints may contribute to the poor prognosis of patients with high-risk scores.

### 2.6. Screening for Potentially Effective Molecules Targeting Prognostic Genes of Glioma

**Figure 8:**
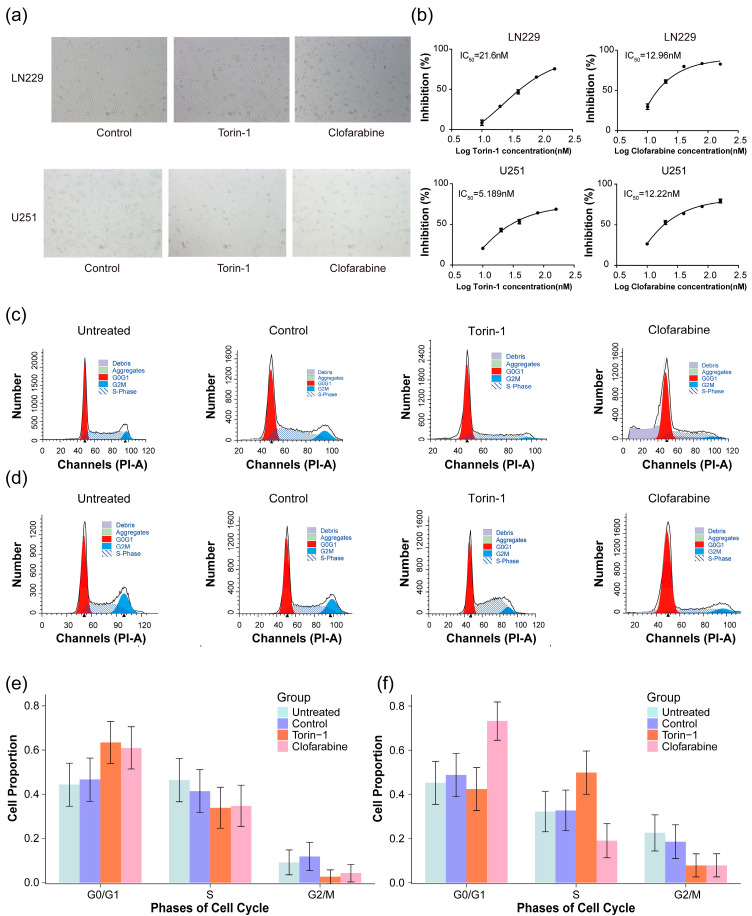
Effect of torin-1 and clofarabine on cell viability and cell cycle in glioblastoma (GBM) cells. (**a**) The images of LN229 (top) and U251 cells (bottom) treated with torin-1 and clofarabine; (**b**) the curves of inhibition and IC_50_ values of torin-1 and clofarabine for LN229 (top) and U251 (bottom) cells. The cells were determined by CCK8 assay after treatment of the drugs for 48 h; (**c**) flow cytometry analysis for cell cycle distribution of LN229, and (**d**) that of U251 cells across different groups, including untreated, control, and perturbation with torin-1 and clofarabine. Cells were treated for 24 h with 3 μM of clofarabine and torin-1, respectively; proportions of cells in different cell cycle phases with 95% confidence intervals (*CIs*) across different groups for (**e**) LN229 and (**f**) U251 cells. The whole cycle for each group was assumed as 100%, with experiments conducted in triplicate.

Drug repurposing was conducted based on the 11 prognostic genes for glioma and the LINCS L1000 platform using the methodology reported in the study [21]. Briefly, the method could screen the drugs that have the potential to reverse cancer-related gene expression and calculate a reversal score for each drug. Sorted by reversal scores for all cases of drugs in the L1000 dataset (a total of 60,510 cases), the drugs with high reversal scores have high potency to reverse the expression of 11 genes and are considered to have high efficacy in the treatment of glioma. Two drugs, torin-1 and clofarabine, were selected for testing their anti-GBM activity due to their high ranks in the list of drugs (20/60,510, 24/60,510, respectively) according to their reversal scores. Subsequently, the dose-dependent effects of torin-1 and clofarabine on the viability of glioma cells were assessed in the LN229 and U251 cell lines, respectively. In LN229 cells, we observed that torin-1 and clofarabine have a significant inhibitory effect on the cells, and the half-maximal inhibitory concentration (IC_50_) values were 21.6 nM and 12.96 nM, respectively (Figure 8a,b, top). In U251 cells, the consistent inhibition of cell viability was observed, and the IC_50_ values for torin-1 and clofarabine were 5.189 nM and 12.22 nM, respectively (Figure 8a,b, bottom). 

Given that the 11 prognostic genes displayed significant correlations with cell cycle pathways (Figure 3b), we conducted a flow cytometry analysis to investigate whether the two drugs influenced cell viability by modulating cell cycle progression. The results for LN229 and U251 cells are presented in Figure 8c,d. A chi-square test was performed to compare the proportions of cell populations in each cell cycle phase—G0/G1, S, and G2/M—between the control and drug-treated groups for both cell lines. The results indicate significant differences in the distribution of cell populations for cell cycle phases across all comparisons (Table 1). Furthermore, torin-1 led to an increase in S phase cells in U251 and prolonged the G0/G1 phase in LN229, while clofarabine induced obvious cell cycle arrest in LN229 and U251 cells, characterized by an increase in G0/G1 phase cells (Figure 8e,f). These results indicate that torin-1 and clofarabine may exert inhibitory effects by preventing cells from progressing into the G2/M phase and inhibiting cell proliferation.

## 3. Discussion

**Figure 9:**
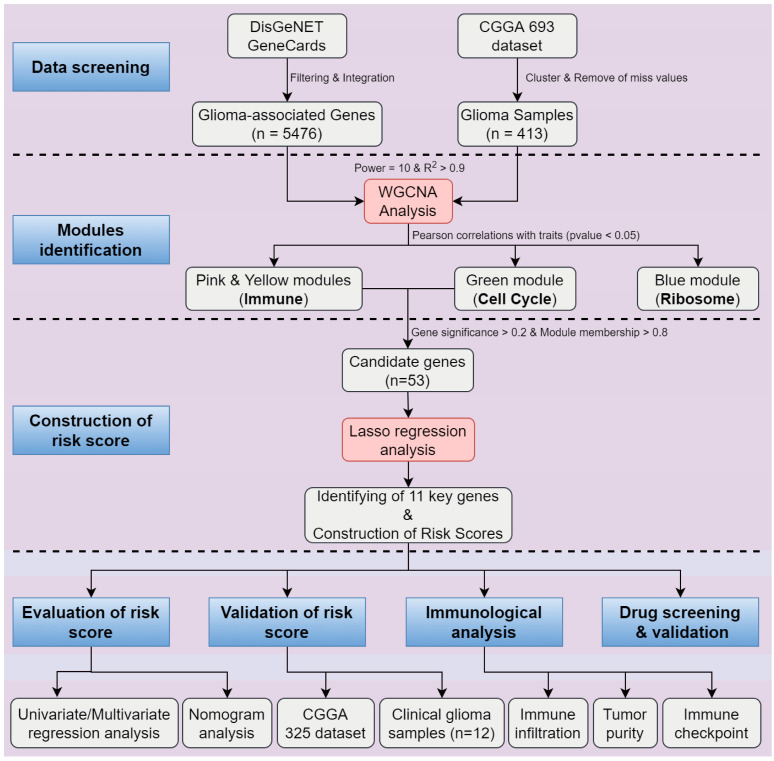
The flowchart of the whole study. The main procedure sequentially comprises six components: (1) Data Screening and Modules Identification, (2) Construction of Risk Score, (3) Evaluation of risk score, (4) Validation of risk score, (5) Immunological analysis, (6) Drug screening and Validation.

With rapid advancement in the basic and clinical research of gliomas, there has been considerable progress in the treatment of brain gliomas. However, improvement of survival time for glioma patients is still an extremely serious challenge due to the obscure pathogenesis, lack of precise diagnostic methods, and effective medicines. Since the combination of histopathological and molecular features was required for the classification and diagnosis of glioma in the 2016 World Health Organization Classification of Tumors of the Central Nervous System (2016 CNS WHO) [5], molecular features in the diagnosis and prognosis for glioma patients have been widely accepted. Undoubtedly, the identification of key genes and the building of prognostic models based on the large public database for glioma, such as the Chinese Glioma Genome Atlas (CGGA) and the Cancer Genome Atlas (TCGA) database, has become increasingly appealing. Recently, to more accurately assess the prognosis of glioma patients, many prognostic models based on the identification of marker genes have been developed. However, there is currently no widely accepted and applied prognostic model [11]. In this study, we have conducted WGCNA analysis based on the CGGA 693 dataset, a transcriptome dataset containing 693 samples, and identified four modules significantly associated with clinical traits for glioma. These modules are significantly enriched in the immune system, cell cycle, and ribosome biogenesis. Upon LASSO regression analysis, 11 key genes were identified. A risk score model was then constructed based on the 11 genes, which can effectively predict risk scores for individual patient with glioma and assess the prognosis. The risk score has been demonstrated to be an independent prognostic factor through both univariate and multivariate regression analysis. Using data from the CGGA 325 dataset and a separate group of 12 glioma patients with varying overall survival (OS) durations, the risk score proved to be an effective tool for assessing patient prognosis. The analysis procedure for this whole study was summarized in Figure 9.

Through WGCNA, LASSO, and multivariate Cox regression analysis, we selected 11 genes from the pink, yellow, and green modules as risk prediction indicators. KIF14, ASPM, CCR5, GINS4, POLD3, TLR2, KIF2C, KPNA2, ITGB2, PLOD1, and CTSZ were highly correlated with the overall survival of glioma patients. The total survival period of glioma patients, LGG, or GBM patients with high-risk scores was significantly lower than that of low-risk score individuals. Even after adjusting for confounding factors such as clinical traits, gene mutations, and 1/3/5-year survival periods, the risk score remained an independent prognostic factor for glioma. We conducted GO functional analysis and pathway enrichment analysis on these key genes and found that they mainly cluster in functions related to the immune system processes, DNA replication, cell cycle, and division, indicating their potential roles in enhancing immune evasion and cell proliferation in gliomas. The kinesin superfamily genes (KIFs) play crucial roles in the cell cycle and have been shown to be involved in chromosome and spindle movements during mitosis and meiosis. KIF14 is highly expressed in gliomas and is associated with higher mitotic and Ki67 indices, as well as lower patient survival rates [22]. Knocking down KIF14 can reduce cell proliferation and invasion capabilities, induce apoptosis, and inhibit glioma growth [23], suggesting that KIF14 may be a potential target for HGBT treatment [24]. KIF2C is involved in regulating cell mitosis and the repair of double-strand DNA breaks in cancer cells. Research by Tu et al. found that KIF2C is highly expressed in primary and recurrent gliomas and is associated with shorter patient survival, potentially serving as a prognostic biomarker for GBM [25]. Studies have shown that ASPM expression is associated with the WHO grade of gliomas, with higher expression in GBM and recurrent tumors. ASPM is involved in the cell cycle and microtubule stability in gliomas [26,27], suggesting that ASPM depletion may mimic microtubule destabilizers; however, specific ASPM inhibitors have not been discovered yet. CCR5 is highly expressed in glioblastomas and can trigger the PI3K/AKT pathway to promote proliferation, induce polarization of TAMs and immune suppression and facilitate the maintenance of glioma stem-like features [28]. The GINS (Go-Ichi-Ni-San) complex plays a crucial role in DNA replication and the cell cycle. The GINS complex consists of four subunits encoded by the GINS1, GINS2, GINS3, and GINS4 genes. While there is abundant research reporting high expression of GINS2 in various tumors, including gliomas, promoting tumor cell proliferation and migration, inhibiting apoptosis, and regulating the cell cycle, it can serve as a new diagnostic marker and therapeutic biomarker for tumors [29]. Although there are no reports on the expression and function of GINS4 in gliomas, as a member of the GINS complex, GINS4 plays an important role in cell cycle regulation. DNA polymerase delta (POLD) is a heterotetramer composed of a catalytic subunit and three accessory subunits. Abnormal expression and gene mutations of POLD can lead to tumorigenesis. Mutations in the POLD1 and POLD3 genes increase the risk of CRC and other malignant tumors [30]. Dysregulation of POLD3 expression can affect genomic stability, promote cell differentiation and proliferation, and serve as a prognostic factor predicting the survival rate of LGG patients [31,32]. Recent studies have reported that POLD/POLE mutations are associated with the immune response to CRC [33]. Toll-like receptors (TLRs) play a crucial role in innate immunity, and tumor-associated macrophages play a significant role in the tumor microenvironment. Activation of TLR2 can promote M2 polarization of TAMs to enhance tumor growth. Additionally, TLR2 activation can upregulate MMP1 expression in microglia and gliomas, promoting tumor growth and migration [34]. KPNA2 is highly expressed in various malignant tumors. In gliomas, KPNA2 can promote tumor growth and metastasis by inhibiting the activation of the HIPPO signaling pathway and mediating TP53 nuclear translocation to promote EMT [35,36]. ITGB2 is a common and important gene in glioma immunity and stromal infiltration. Tumor infiltration analysis indicates that ITGB2 is associated with immune cells such as dendritic cells, macrophages, monocytes, neutrophils, and B cells. ITGB2 is considered a potential target for primary GBM [37]. Cathepsin Z (CTSZ) is a protease that not only has protease activity but also interacts with integrins. Although there is limited research on the role of CTSZ in gliomas, studies in pancreatic neuroendocrine tumors have shown that intracellular CTSZ in tumor cells can promote proliferation, while both intracellular CTSZ in tumor cells and CTSZ secreted by TAMs can promote tumor invasion [38]. CTSZ may have similar functions in gliomas and the microenvironment. Overall, the functions of these genes in tumors, most of which have been well established in gliomas, suggest that KIF14, ASPM, CCR5, GINS4, POLD3, TLR2, KIF2C, KPNA2, ITGB2, PLOD1, and CTSZ can be considered key prognostic factors for the overall survival of glioma patients.

In addition, we established a prognostic scoring model based on 11 hub genes to predict risk in both the CGGA dataset containing 325 patients and 12 clinical samples of gliomas. We found that the expression of these 11 genes in WHO IV is significantly higher than in WHO II and WHO III, and the risk score is closely associated with age, grading, IDH mutation, and 1p/19q loss. However, this predictive model cannot effectively distinguish between GBM patients with long or short progression-free survival. Considering the high malignancy and rapid progression of GBM, as well as its significant tumor heterogeneity and short overall survival, existing predictive models struggle to provide accurate predictions for GBM. Future research focusing on prognostic models based on different pathological subtypes of GBM will be beneficial for survival and prognosis predictions in GBM.

The tumor microenvironment has a complex relationship with the onset, progression, and metastasis of gliomas. Unlike other tumors, gliomas have a unique immune microenvironment. Due to the presence of the blood–brain barrier, tumor-infiltrating lymphocytes (TILs) are scarce in gliomas, with the microenvironment mainly infiltrated by microglia and tumor-associated macrophages (primarily M2-type macrophages). M2-type macrophages can produce growth factors, activate tissue repair and angiogenesis, suppress adaptive immunity, enhance immune escape, and promote tumorigenesis. TILs primarily consist of anti-tumor cells, including CD8+ and CD4+ T cells (helper T cells), with CD8+ T cells associated with a good prognosis. The TME also contains Treg cells, which participate in tumor immune suppression processes and are associated with poor prognosis [39]. Previous studies have reported that CCR5, TLR2, ITGB2, and CTSZ, among the 11 hub genes, are involved in TAM polarization and immune suppression in gliomas. In this study, we found that the risk score significantly influences the adaptive immunity in gliomas and is strongly correlated with TAMs. Patients with high-risk scores have anti-tumor-related TILs in the TME predominantly in a dormant state, while patients with low-risk scores have higher levels of anti-tumor-related TILs in the TME. Furthermore, the risk score is positively correlated with stromal and immune scores and negatively correlated with tumor purity. These results suggest that our constructed risk prediction model may predict the immune-suppressive environment in glioma patients. Immune checkpoint inhibitors are widely used in cancer treatment and have shown good efficacy in various cancers; however, they have shown poor efficacy in glioma treatment [40]. Our risk score indicates a significant positive correlation between immune checkpoint-related molecules, such as PD-1/PD-L1, CTLA-4/CD80, TIM-3/LGALS9, LAG3/HLA-DRA, and the risk score, indicating that higher risk scores are associated with higher immune checkpoint expression, stronger immune evasion capabilities, and potentially poorer response to the immune checkpoint blockade (ICB) therapy. Therefore, our established risk score may be useful for evaluating the prognosis of glioma patients after receiving ICB treatment.

The conventional treatment methods for gliomas include surgery, chemotherapy, and radiotherapy; however, the prognosis for malignant glioma patients remains discouraging, with conventional therapies showing only limited improvements for glioma patients [41]. Malignant gliomas exhibit genetic heterogeneity, and a single therapy is not sufficient to address clinical issues. The 11 hub genes included in our prognostic model are significantly associated with the clinical traits of gliomas. We conducted small molecule inhibitor screening for these 11 hub genes and found that clofarabine and torin-1 exhibit high inhibitory activity against two glioma cell lines, LN229 and U251, blocking cells in the G0/G1 phase, inhibiting the proportion of cells in the G2 phase, and affecting the cell cycle process, thereby inhibiting cell proliferation. This indicates that these hub genes can serve not only as prognostic indicators but also as targets for glioma treatment.

## 4. Materials and Methods

### 4.1. Data Acquisition and Reprocessing

Two datasets from two distinct Chinese glioma cohorts (the 693 and 325 cohorts) were downloaded from the Chinese Glioma Genome Atlas database (CGGA, http://www.cgga.org.cn (accessed on 4 July 2023)) [12], named CGGA 693 and CGGA 325 datasets, respectively, which included the raw expression matrix and corresponding clinical information. In our study, the CGGA 693 dataset was used as the primary dataset for analysis, while the CGGA 325 dataset was used as one of the validation datasets. For the CGGA 693 dataset, we first performed cluster analyses using t-distributed stochastic neighbor embedding (tSNE) and uniform manifold approximation and projection (UMAP) methods and achieved two clustering groups. We selected the primary group of 626 samples as the object for the study. After removing missing clinical data, the primary group included a total of 413 glioma samples. The statistics of clinical characteristics for the datasets with 413 samples and the CGGA 325 dataset are shown in Table S4. The expression matrix for low-grade glioma (LGG) and glioblastoma multiforme (GBM), comprising raw counts and fragments per kilobase per million mapped fragments (FPKM), along with the associated clinical information, were obtained from the Cancer Genome Atlas (TCGA, https://www.cancer.gov/tcga (accessed on 4 July 2023)). The raw counts in all datasets were normalized using the Trimmed Mean of M-values (TMM) algorithm from the edgeR package (v3.36.0) in R (v4.1.3) [42].

### 4.2. Collection of Genes Associated with Glioma

To obtain relatively reliable glioma-related genes, the DisGeNET database (https://www.disgenet.org/ (accessed on 17 November 2023)) was initially consulted to compile genes linked to Glioma (C0017638) and malignant glioma (C0555198), respectively [43]. After eliminating duplicates, a total of 3202 genes were obtained. Subsequently, the term “Glioma” was used to search the Genecards database (https://www.genecards.org/ (accessed on 17 November 2023)), yielding 9446 glioma-related genes, in which filtering is carried out based on a criterion where only genes with relevance scores greater than the median score (0.75) were retained, resulting in 4621 genes. By integrating genes from both databases, a total of 5476 unique genes with entry IDs were compiled (Table S1). These genes will be utilized for subsequent analyses.

### 4.3. Weighted Correlation Network Analysis (WGCNA) and Identification of Modules

To identify the modules of co-expressed genes associated with clinical traits for glioma, we employed the WGCNA method as described by Langfelder and Horvath [44], which was performed using the WGCNA package (v1.72.1) in R. 

After checking excessive missing values and identification of outliers, a soft thresholding power of *β* = 10 was selected for the dataset (a matrix of 5476 (genes) × 413 (samples)) by the function pickSoftThreshold() of WGCNA package to ensure scale-free topology, R^2^ > 0.9, based on the criterion of approximate scale-free topology (Figure S2a).

The WGCNA::blockwiseModules() function was used with the following settings for co-expression network construction and module detection in a one-step way: soft threshold power (*β*) = 10, minimum module size of 30, merge cut height of 0.5. Briefly, a co-expression similarity matrix *S_ij_* was constructed by calculating Pearson’s correlation coefficients between gene pairs of the collected glioma-related genes (5476) across the 413 glioma samples. The co-expression similarity matrix *S_ij_* was defined as the absolute value of the correlation coefficient between the expression profiles of gene *i* (*x_i_*) and gene *j* (*x_j_*). The formula of *S_ij_* is as follows:

$$S_{ij}=|cor(x_{i},x_{j})|$$

Subsequently, the similarity matrix was transformed into a weight adjacency matrix (*a_ij_*) using the selected soft thresholding power (*β*), and the formula was shown as follows:

$$a_{ij}=s_{ij}^{\beta }$$

The adjacency matrix was then converted into a topological overlap matrix (TOM) with TOM similarity and its dissimilarity (dissTOM). Hierarchical clustering on the dissTOM was performed to group genes into modules. A dynamic tree cut algorithm was conducted on the dendrogram for identifying co-expression gene modules, with a minimum size requirement of 30 and merge cut height of 0.5. The first principal component of the expression of each module was summarized as module eigengenes (MEs). Pearson’s correlations between MEs and clinical traits of gliomas were computed, and modules exhibiting significant correlations (*p*-value < 0.05) with clinical traits such as IDH and 1p/19q status were singled out for further analysis. The candidate genes in the selected modules are defined as having high significance with the traits (gene significance, GS) as well as high intramodular connectivity (module membership, MM), representing the correlation between gene expression and *MEs*. The candidate genes in the module were screened out by setting GS > 0.2 and MM > 0.8 as a threshold.

### 4.4. Gene Function Enrichment Analysis

Gene ontology (GO) and reactome pathway analysis were performed by online DAVID (https://david.ncifcrf.gov/home.jsp (accessed on 5 March 2024)) [45] with default parameters (Count: 2 and EASE Score: 0.1), and terms with *p*-value < 0.05 were considered to be statistically significant. Gene set enrichment analysis (GSEA) was performed using the GSEA Preranked method in GSEA software (v4.1.0) with default parameters employed as follows: Number of permutations: 1000, Collaps/Remap to gene symbols: No_Collapse, Min size: 15, Max size: 500. The significance of the enrichment was assessed at *p*-value < 0.05 and FDR < 0.25 [46,47].

### 4.5. Construction of Glioma-Related Prognostic Signature

A total of 53 genes exhibiting a significant correlation with IDH and 1p/19q status within the Immune and Cell cycle modules were utilized in the least absolute shrinkage and selection operator (LASSO) Cox regression analysis to determine specific coefficients for each association. LASSO Cox regression is a method for variable selection and shrinkage in the Cox proportional hazards model proposed by Tibshirani et al. [48]. LASSO Cox regression analysis was carried out using the glmnet package (v4.1.6) in R. Briefly, we generated a Surv object using the survival package (v3.55) in R based on the survival time and censoring information for 413 patients, which was used as the response variable in the function glmnet() of glmnet package. The expression matrix for 53 screened genes across 413 patients was used as predictor variables. Next, we ran the function glmnet() with the parameters family = “Cox” and alpha = 1 to fit the LASSO regression model. 

To determine what value to use for lambda, we performed 10-fold cross-validation using the function cv.glmnet() with parameter k = 10 and identified the lambda value that produces the lowest test mean squared error (MSE). The lambda value that minimized the test MSE turned out to be 0.04067 in our study.

Lastly, we analyzed the final model produced by the optimal lambda value to obtain the coefficient estimates for this model. For the genes with no coefficients in predictor variables, this means it was completely dropped from the model because it was not influential enough. Then, 11 prognostic genes and corresponding coefficients were achieved to develop a glioma-related prognostic signature. The risk score for each patient was computed using the following formula:

$$RiskScore=\sum_{i=1}^{n}{expr}_{gene\left(i\right)}\times {coeff}_{gene(i)}$$

In this formula, n represents the number of genes selected by the LASSO method, *expr_gene_*_(*i*)_ is the expression value of *gene*(*i*), and *coeff_gene_*_(*i*)_ is the Cox coefficient of *gene*(*i*).

### 4.6. Protein-Protein Interaction (PPI) Analysis

The key genes meeting the criteria of gene significance (GS) for IDH status > 0.2 and module membership (MM) > 0.8 within the four significantly identified modules were used as input for the STRING database (https://cn.string-db.org/ (accessed on 4 March 2024)), a resource for predicting functional associations among proteins, to perform protein–protein interaction network analysis. The MCODE plugin (v2.0.0) in Cytoscape (v3.1.0) was used to detect densely connected regions within the resulting extensive protein–protein interaction networks. All network visualizations were performed by using Cytoscape.

### 4.7. Prognostic Model Based on Clinical Traits and Risk Scores

Univariate survival analyses were performed on the risk scores and clinical traits of glioma patients using univariate Cox proportional hazard models. Subsequently, a multivariate Cox model was executed utilizing the same features to determine if each feature could serve as an independent prognostic variable for glioma patients.

A nomogram model was constructed using the ‘rms’ package (v6.8.0) in R software to predict clinical outcomes for glioma patients by integrating risk scores with four clinical traits(Age, Grade, IDH status, 1p/19q status). To evaluate the effectiveness of this nomogram model in predicting the 1-year, 3-year, and 5-year survival rates of patients, a series of calibration plots and a receiver operating characteristic (ROC) curve were generated.

### 4.8. Glioma Tissues and RNA Sequencing

A total of 12 patients diagnosed with high or low-grade glioma (4 LGG and 8 GBM) were selected from the archives of Tianjin Medical University Cancer Institute and Hospital (TMUCIH). Frozen tumor samples were collected from these patients for mRNA next-generation sequencing using the Illumina-HiSeq2000/2500 platform. All patients were newly diagnosed and had not undergone chemo-or radiotherapy before surgery. Patients received concurrent radiotherapy, temozolomide (TMZ) chemotherapy, and TMZ sequential chemotherapy after surgery until tumor recurrence. The patients were monitored for a period ranging from 4 to 132 months, and unfortunately, all patients had passed away by the end of the study. This study was thoroughly reviewed and approved by the Ethics Committee of TMUCIH.

### 4.9. Immunohistochemical Staining

The tumor samples of patients were processed into 5 μm tissue sections after formalin-fixation and paraffin embedding. The sections underwent deparaffinization in xylene and rehydration using a descending series of ethanol. Subsequently, tissue antigens were repaired by microwave heating, followed by incubation with 10% normal goat serum to block nonspecific reactions at room temperature for 10 min. Primary antibodies against KPNA2 (1:200; Proteintech, Wuhan, China), ASPM (1:200; Proteintech, China), CCR5 (1:100; Proteintech, China), KIF2C and KIF14 (1:100; Proteintech, China) were applied separately and incubated overnight at 4 °C, and the biotin-labeled goat anti-mouse/rabbit IgG and streptavidin-peroxidase (UltraSensitiveTM SP IHC Kit; Fuzhou Maxim Biotech, Fuzhou, China) were subsequently used. After washing, the sections were then developed using a diaminobenzidine substrate.

### 4.10. Immune Cell Infiltration Analysis

To quantify the infiltration of 28 types of immune cells in each glioma sample, ssGSEA analysis was performed using the GSVA packages (v1.42.0) in R [49]. The ssGSEA enrichment score was utilized as the measure of immune cell infiltration in each sample. Gene signatures for each immune cell type were adopted from a previous study. The CIBERSORTx (http://cibersort.stanford.edu (accessed on 2 April 2024)) method was performed to characterize cell composition based on gene expression profiles in previous studies.

### 4.11. Estimation of Stromal and Immune Cells

The Estimation of Stromal and Immune cells in MAlignant Tumours using the Expression data (ESTIMATE) tool (1.0.13) was employed to calculate the immune score and stromal score [50,51], representing the degree of infiltration of immune cells and stromal cells, respectively. The ESTIMATE score was then produced by integrating the stromal scores and the immune scores. The formula for tumor purity is as follows [51]:

Tumor purity=cos⁡(0.6049872018+0.0001467884×ESTIMATE score)

### 4.12. Drug Screening Based on Prognostic Genes

Expression regulatory profiles (upregulation or downregulation) of 11 glioma prognostic genes were analyzed to identify potential therapeutic drugs capable of reversing the expression of these genes by the method reported in the study [21], using the LINCS L1000 database [52]. The LINCS L1000 database offers gene expression profiles induced by over 10,000 compounds, shRNAs, and kinase inhibitors using the L1000 platform. Briefly, the method could screen the drugs that have the potential to reverse cancer-related gene expression and calculate a reversal score for each drug. Sorted by reversal scores for all cases of drugs in the L1000 database, the drugs with high reversal scores have high potency to reverse the expression of 11 genes and were considered to have high efficacy in the treatment of glioma.

### 4.13. Cell Culture and Drug Perturbation

Human glioma cell lines LN229 were sourced from the American Type Culture Collection (ATCC), while the U251 cell line was obtained from Sigma. All cell lines were cultured in Dulbecco’s modified Eagle’s medium (DMEM) (Gibco, Carlsbad, CA, USA) supplemented with 10% fetal bovine serum (Sigma, St. Louis, MO, USA) and 1% penicillin/streptomycin (Gibco, Carlsbad, CA, USA) and maintained at 37 °C in a humidified atmosphere with 5% CO_2_.

To assess the cytotoxic effect of compounds, LN229 and U251 cell lines were subjected to a CCK8 assay. Cells were seeded into 96-well plates at a density of 2–5 × 10^3^ cells/well and allowed to adhere and grow overnight. After treatment with different concentrations of clofarabine and torin-1 (MedChemExpress, Shanghai, China) for 48 h, cell viability was detected by adding 10 µL CCK8 reagent (GLPBIO, Montclair, CA, USA) to each well and another 1 h normal culture and the optical density (OD) was measured at 450 nm using a Microplate Reader (Bio-Rad, Hercules, CA, USA). Cell proliferation inhibition rates were calculated to assess the impact of different treatments on cell viability, using the formula: cell proliferation inhibition rate = 100% × [mean OD value of control group − mean OD value of treatment group]/mean OD value of control group.

### 4.14. Experiment and Analysis of Cell Cycle

The cell cycle was assessed using the Cell Cycle Staining Kit (MULTI SCIENCES). LN229 cells and U251 cells were seeded into 6cm dishes with 2–5 × 10^6^ cells/well and allowed to adhere and grow overnight. Subsequently, the cells were treated with 3 μM of the respective drugs. After a 24-hour incubation period with the drugs, the cells were collected and centrifuged to remove the supernatant, and the cell pellet was resuspended and washed with PBS (Gibco). Single-cell suspensions were then stained with 1 mL of propidium iodide (PI) staining solution and 10 μL of permeabilization solution at 4 °C for 30 min in a final volume of 200 μL. The labeled cells were analyzed using flow cytometry, and the results were presented as the percentage of cells in each phase of the cell cycle. The distributions of cell populations in cell cycle phases between the control and drug-treated groups were assessed using the Chi-square test via the online tool Epitools (https://epitools.ausvet.com.au/chisq (accessed on 27 August 2024)).

### 4.15. Statistical Analysis

All statistical analyses and figures were performed using R language (version 4.1.3). Student’s t-test or Wilcox test was used to determine the significance of differences between groups. Kaplan−Meier survival analysis was used to assess the statistical significance between two groups using R packages (survival and ggplot2). Pearson or Spearman correlation was used to measure the correlation between two variables, which were calculated by function cor.test() in R or JASP tool [53]. The details of the source and version of the algorithms and software involved in this study are listed in Table S5. 

## 5. Conclusions

In conclusion, we constructed a gene co-expression network for gliomas and identified risk assessment characteristics of 11 hub genes. This prognostic model demonstrates good efficiency in predicting the prognosis of glioma patients. The high-risk scores of these characteristics predict immune suppression and evasion capabilities in gliomas, potentially serving as prognostic indicators for immune checkpoint blockade therapy. Interestingly, we identified two small molecule inhibitors targeting the risk feature genes, demonstrating effective inhibition of glioma cell activity and cell cycle progression, providing new insights for glioma treatment.

## Figures and Tables

**Table 1:** Chi-square test results for the proportion of cell populations in each cell cycle phase (G0/G1, S and G2/M) between the control and drug-treated groups for the LN229 and U251 cell lines.

	LN229 Cells		U251 Cells	
	Control and Torin-1	Control and Clofarabine	Control and Torin-1	Control and Clofarabine
Chi-square statistic	9.110	6.042	8.342	12.849
Degree of freedom	2	2	2	2
*p*-value	0.011	0.049	0.015	0.002

## Data Availability

All data generated or analyzed during this study are included in this published article.

## Supplementary Materials

The following supporting information can be downloaded at https://www.mdpi.com/article/10.3390/ph17101295/s1, Figure S1: Clustering and survival analysis for samples of CGGA 693 dataset; Figure S2: WGCNA analysis for 413 high-quality glioma patients from the CGGA 693 dataset; Figure S3: The distribution of partial gene expression within the blue modules identified by WGCNA in both non-codel and codel groups of samples; Figure S4: Kaplan–Meier survival analysis for patients between high-risk and low-risk patients across various datasets; Figure S5: Experimental validation of some prognostic genes by using Immunohistochemical test; Table S1: Integration of genes associated with glioma from DisGeNET and GeneCards; Table S2: MCODE analysis results; Table S3: Survival analysis of 96 candidate genes from 4 identified modules in CGGA 693 and CGGA 325 dataset; Table S4: Clinical traits of glioma patients from CGGA database; Table S5: Details of algorithms or software used in this study.

## Abbreviations

WGCNA	Weighted Correlation Network Analysis
CGGA	Chinese Glioma Genome Atlas
TCGA	The Cancer Genome Atlas
LASSO	Least Absolute Shrinkage and Selection Operator
OS	Overall Survival
LINCS	The Library of Integrated Network-Based Cellular Signatures
GBM	Glioblastoma Multiforme
WHO	World Health Organization
LGG	Lower-grade Gliomas
HGG	High-grade Gliomas
MGMT	O6-methylguanine-DNA Methyltransferase
PTEN	Phosphatase and Tensin Homolog
TP53	Tumor Protein p53
EGFR	Epidermal Growth Factor Receptor
MET	MET Tyrosine Kinase
IDH	Isocitrate Dehydrogenase (gene)
H3K27M	Lysine 27-to-Methionine Mutation in Histone H3
KPS	Karnofsky Performance Status
TMM	Trimmed Mean of M-values
tSNE	t-Distributed Stochastic Neighbor Embedding
UMAP	Uniform Manifold Approximation and Projection
PRS	Primary-Recurrent-Secondary
MM	Module Membership
GS	Gene Significance
MCODE	Molecular Complex Detection
GO	Gene Ontology
BP	Biological Process
HR	Hazard Ratio
ROC	Receiver Operating Characteristic Curve
GSEA	Gene Set Enrichment Analysis
ssGSEA	Single Sample Gene Set Enrichment Analysis
IC_50_	Half-maximal Inhibitory Concentration
ICB	Immune Checkpoint Blockade
FPKM	Fragments per Kilobase per Million Mapped Fragments
TOM	Topological Overlap Matrix
MEs	Module Eigengenes
AUC	Area Under Curve
NES	Normalized Enrichment Score
TMZ	Temozolomide
FDR	False Discovery Rate
