# Supplementary material for: Identification of Key Immune and Cell Cycle Modules and Prognostic Genes for Glioma Patients through Transcriptome Analysis
Source: Pharmaceuticals (Basel). 2024 Sep 28;17(10):1295. doi: 10.3390/ph17101295 (PMC11514598; doi:10.3390/ph17101295)
Supplement: Supplementary file 1 [file pharmaceuticals-17-01295-s001.zip › Supplementary Figures.pdf]

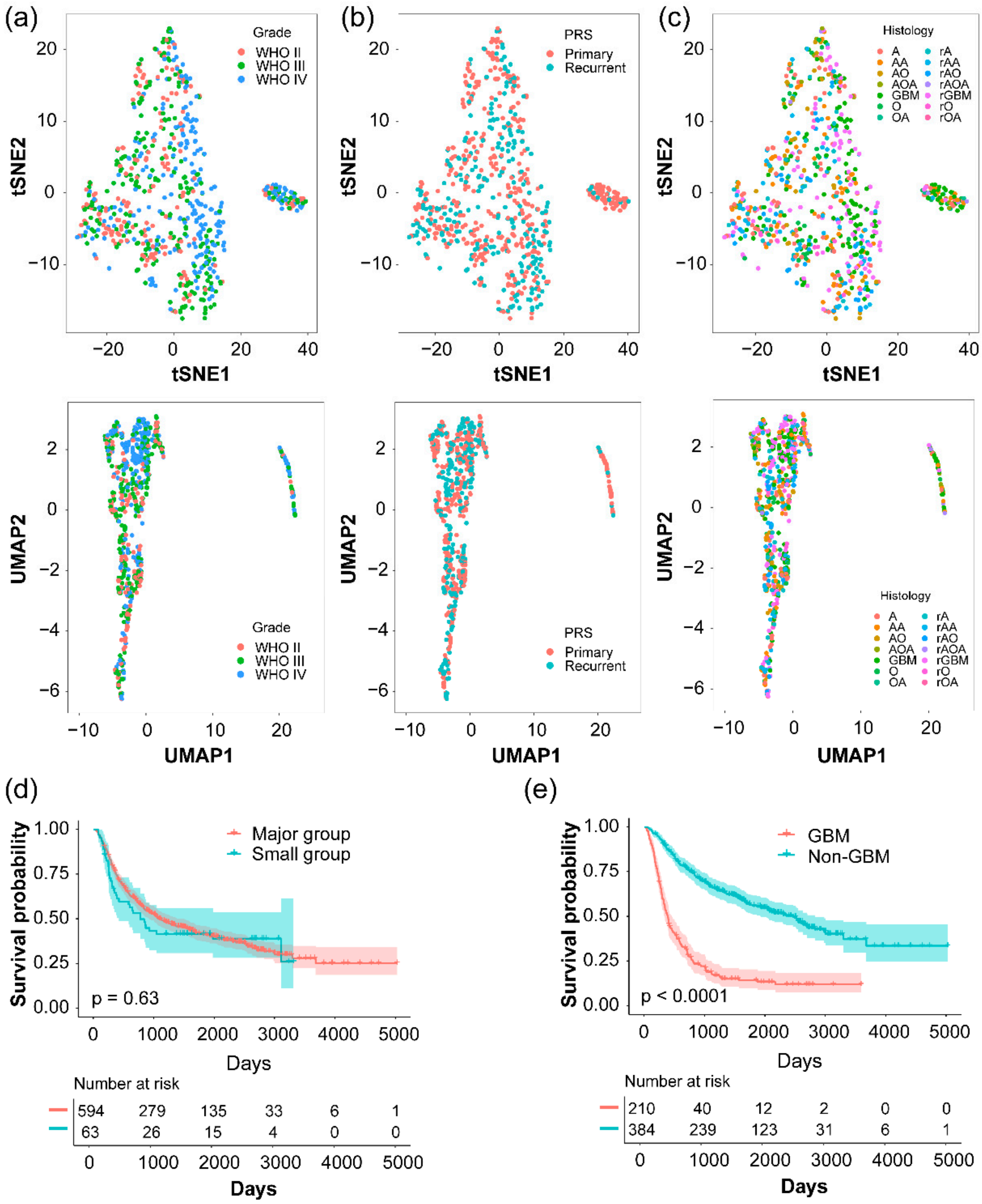

Figure S1. Clustering and survival analysis for samples of CGGA 693 dataset. (**a**) t-distributed stochastic neighbor embedding (tSNE) (top) and uniform manifold approximation and projection (UMAP) (bottom) clustering of 693 samples colored by glioma grade, and (**b**) PRS types, and (**c**) histology types; (**d**) survival analysis between the major and small group of samples indicating no significant differences; (**e**) survival analysis between non-GBM (WHO grade II and III) and GBM (grade IV) within the major group.

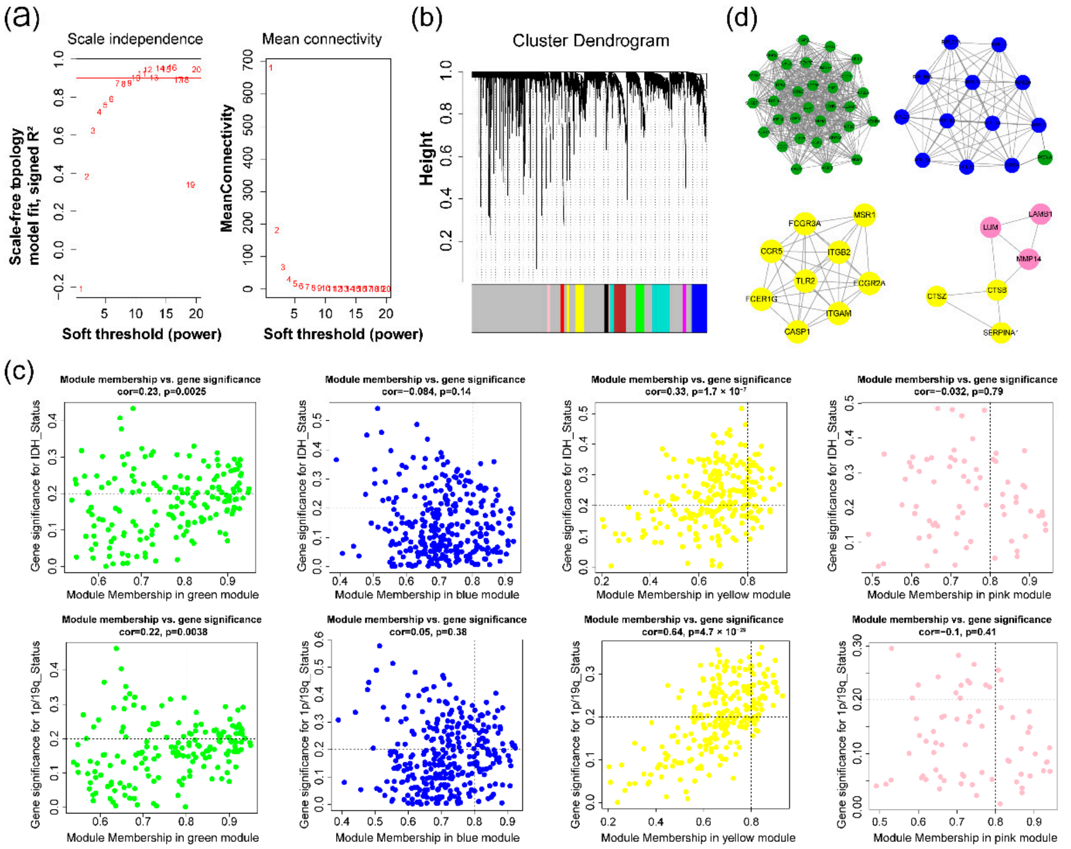

Figure S2. WGCNA analysis for 413 high-quality glioma patients from the CGGA 693 dataset. (a) Soft-threshold (power(β) = 10) and scale-free topology fit index (R^2 = 0.90); (b) clustering dendrograms showing genes with similar expression patterns were clustered into coexpression; (c) scatter plots of the module membership (MM) and gene significance (GS) of candidate gene within green, blue, yellow and pink modules. The top panel coresponding to IDH mutant status for GS and bottom panel corresponding to 1p/19q codel status; (d) the network graph displaying the four highest-scoring clusters as determined by the MCODE algorithm in Cytoscape.

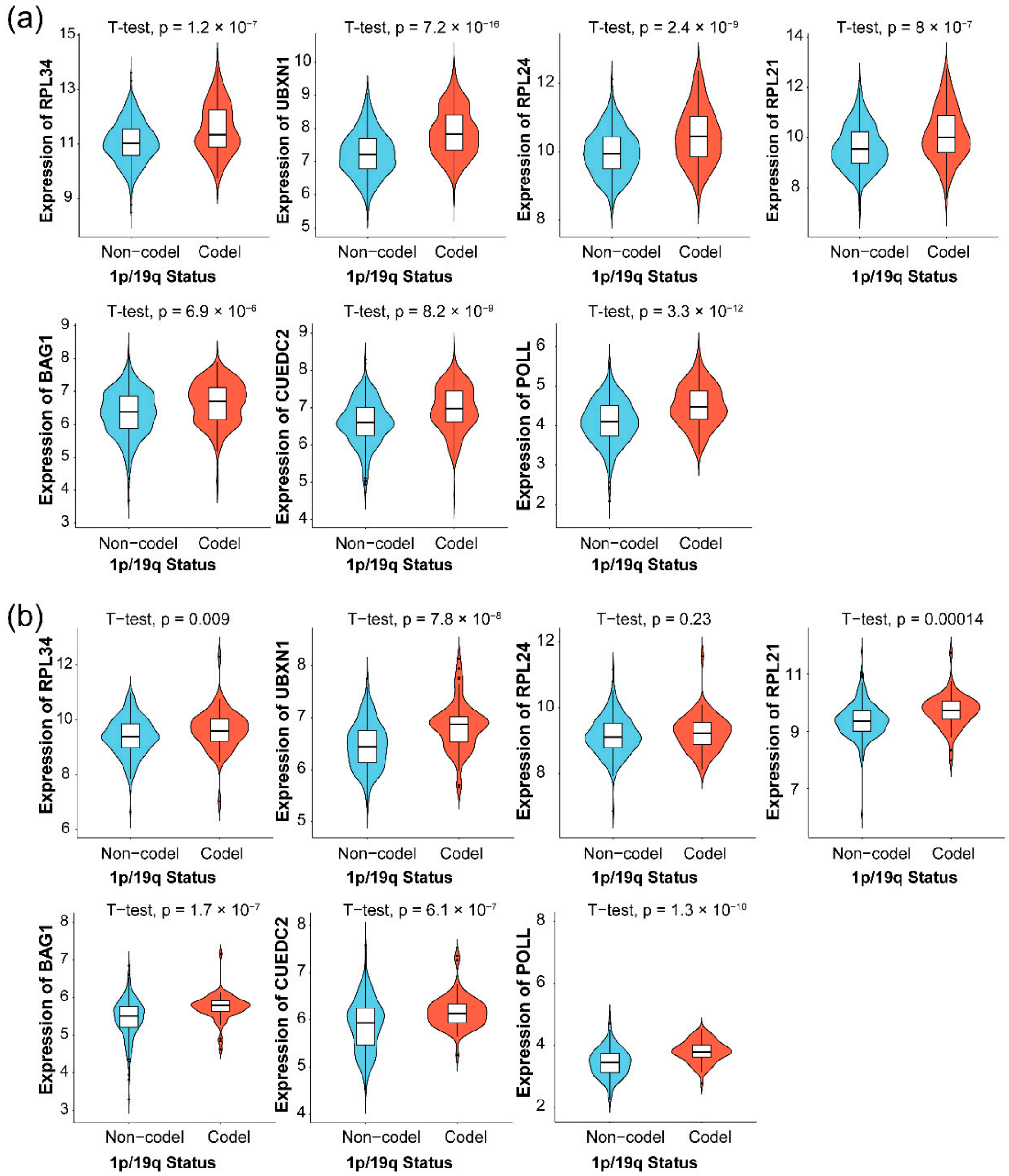

Figure S3. The distributions of partial gene expression within the blue modules identified by WGCNA in both non-codel and codel groups of samples from (a) CGGA 693, and (b) CGGA 325 database.

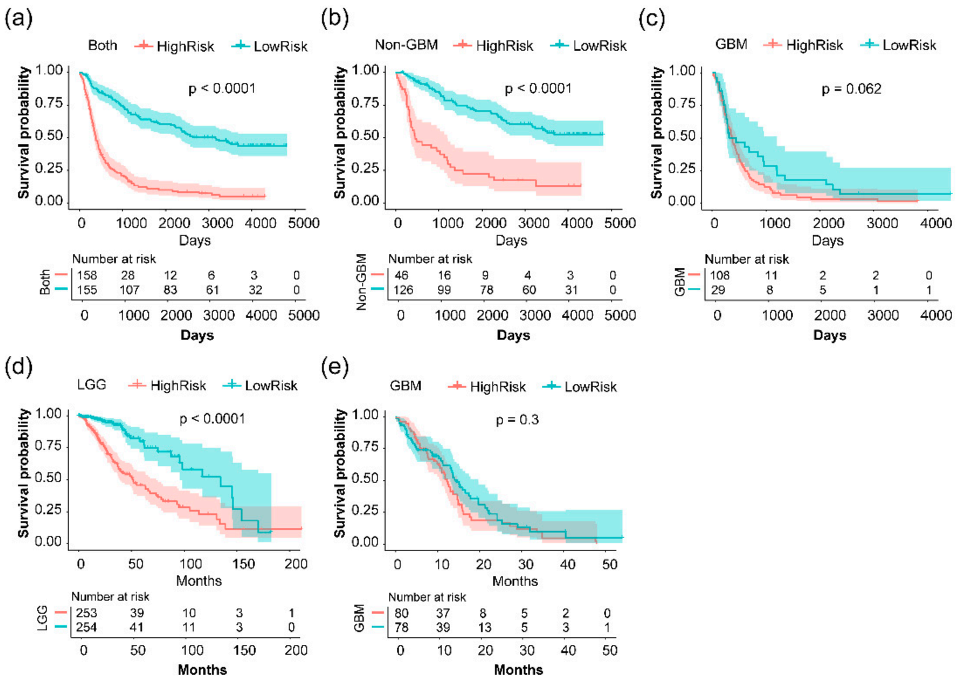

Figure S4. Kaplan–Meier survival analysis for patients between high-risk and low-risk patients across various datasets. (a) All samples from CGGA 325 dataset; (b) a subset comprising patients diagnosed with glioma grade II and III (non-GBM) from the CGGA 325 dataset; (c) a subset comprising patients with glioma grade IV (GBM) from theCGGA 325 datase; (d) all samples from TCGA LGG dataset; (e) all samples from TCGA GBM dataset.

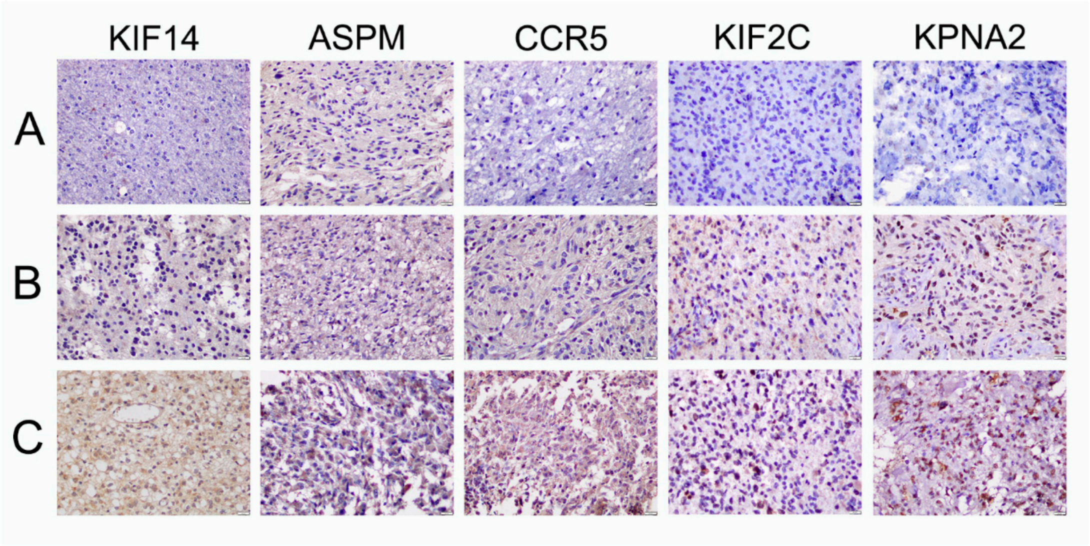

Figure S5. Experimental validation of some prognostic genes by using Immunohistochemical test.
