# Supplementary material for: Identification of Key Immune and Cell Cycle Modules and Prognostic Genes for Glioma Patients through Transcriptome Analysis
Source: Pharmaceuticals (Basel). 2024 Sep 28;17(10):1295. doi: 10.3390/ph17101295 (PMC11514598; doi:10.3390/ph17101295)
Supplement: Supplementary file 1 [file pharmaceuticals-17-01295-s001.zip › Table S4.pdf]

Table S4. Clinical characteristics of glioma patients from CGGA database.

**Characteristics**	**CGGA_325 (N=325)**	**CGGA_693 (N=413)**	**p.overall**
Age	42.9 (12.0)	43.0 (12.4)	0.942
Gender:			0.087
Female	122 (37.5%)	182 (44.1%)	
Male	203 (62.5%)	231 (55.9%)	
PRS:			<0.001
Primary	229 (71.3%)	248 (60.0%)	
Recurrent	62 (19.3%)	165 (40.0%)	
Secondary	30 (9.35%)	0 (0.00%)	
Grade:			<0.001
WHO II	103 (32.1%)	96 (23.2%)	
WHO III	79 (24.6%)	161 (39.0%)	
WHO IV	139 (43.3%)	156 (37.8%)	
Censor:			0.142
Alive	96 (30.4%)	148 (35.8%)	
Dead	220 (69.6%)	265 (64.2%)	
Radiotherapy:			0.889
No	66 (21.3%)	85 (20.6%)	
Yes	244 (78.7%)	328 (79.4%)	
Chemotherapy(TMZ):			<0.001
No	111 (36.5%)	89 (21.5%)	
Yes	193 (63.5%)	324 (78.5%)	
IDH_Status:			0.804
Mutant	175 (54.0%)	228 (55.2%)	
Wildtype	149 (46.0%)	185 (44.8%)	
1p/19q_Status:			1.000
Codel	67 (21.1%)	87 (21.1%)	
Non-codel	250 (78.9%)	326 (78.9%)	
MGMTp_Status:			0.046
Methylated	157 (51.3%)	244 (59.1%)	
Un-methylated	149 (48.7%)	169 (40.9%)	
