# Supplementary material for: Identification of Key Immune and Cell Cycle Modules and Prognostic Genes for Glioma Patients through Transcriptome Analysis
Source: Pharmaceuticals (Basel). 2024 Sep 28;17(10):1295. doi: 10.3390/ph17101295 (PMC11514598; doi:10.3390/ph17101295)
Supplement: Supplementary file 1 [file pharmaceuticals-17-01295-s001.zip › Table S5.pdf]

Table S5. Details of algorithms or software used in this study.

Algorithm	Source	Version	Notes
TMM	R: edgeR package	v3.36.0	R version 4.1.3
tSNE	R: Rtsne	v0.16	R version 4.1.3
UMAP	R: umap package	v 0.2.10.0	R version 4.1.3
WGCNA	R: WGCNA package	v1.72.1	R version 4.1.3
Dynamic Tree Cut	R: WGCNA package	v1.72.1	R version 4.1.3
GSEA	GSEA software	v4.1.0	GSEA version 4.1.0
LASSO	R: glmnet package	v4.1.6	R version 4.1.3
MCODE	Cytoscape: app	v2.0.0	Cytoscape v3.1.0
Univariate Cox Proportional Hazards Model	R: survival package	v3.55	R version 4.1.3
Multivariate Cox Proportional Hazards Model	R: survival package	v3.55	R version 4.1.3
Nomogram Model	R: rms package	v6.8.0	R version 4.1.3
ssGSEA	R: GSVA package	v1.42.0	R version 4.1.3
CIBERSORTx	https://cibersortx.stanford.edu/	Access date: April 2, 2024	--
ESTIMATE	R: estimate package	v1.0.13	R version 4.1.3
JASP	https://jasp-stats.org/	v0.19.0.0	--
